# Can the use of an inclinometer improve acetabular cup inclination in total hip arthroplasty? A review of the literature

**DOI:** 10.1177/1120700020946716

**Published:** 2020-08-04

**Authors:** Bernard H van Duren, Joseph M Royeca, Conor M Cunningham, Jonathan N Lamb, Chris J Brew, Hemant Pandit

**Affiliations:** 1 Leeds Orthopaedic and Trauma Sciences, Leeds Institute of Rheumatic and Musculoskeletal Medicine, University of Leeds, Leeds, UK; 2 Bradford Royal Infirmary, Bradford, UK; 3 Indiana University School of Medicine, West Lafayette, IN, USA

**Keywords:** Acetabular component orientation, inclinometer, operative inclination, radiographic inclination, total hip arthroplasty

## Abstract

**Introduction:**

The angle of acetabular (cup) radiographic inclination is an important measurement in total hip arthroplasty (THA) procedures. Abnormal radiographic inclination is associated with dislocation, edge loading and higher failure rates. Consistently achieving a satisfactory radiographic inclination remains a challenge. Inclinometers have been increasingly used over the last decade. This paper reviews the literature to determine whether using an inclinometer improves the accuracy of acetabular cup inclination in THA.

**Methods:**

A systematic literature search was performed. The following search terms were used: (‘hip’ OR ‘hip replacement’ OR ‘hip arthroplasty’ OR ‘primary hip replacement’ OR ‘THR’ OR ‘THA’ OR ’Acetabular cup Inclination’) AND (‘Inclinometer’). Titles and abstracts were screened for relevance. Both radiographic and operative inclination comparisons were included.

**Results:**

7 studies met the inclusion criteria. 2 were randomised control trials with level I evidence, and the remaining studies were cohort studies with level III/IV evidence. 5 were clinical and 2 experimental. In total there were 16 cohorts: 7 using an inclinometer, 6 freehand, and 3 using MAG techniques. All studies comparing radiographic inclination and 1 of 2 studies comparing operative inclination showed an improvement in the attainment of the optimal inclination. Similarly, the use of an inclinometer showed a reduction in the number of outliers when compared to MAG and freehand techniques.

**Discussion:**

This review demonstrates that using an inclinometer improved the surgeon’s ability to achieve their intended inclination (both operative and radiographic) and reduced the incidence of positioning outside the safe-zone. However, only 2 of the studies were randomised control trials and these resulted in opposing conclusions. Therefore, further studies looking at the use of inclinometers would prove useful in understanding their true benefit.

## Introduction

Total hip arthroplasty (THA – also known as total hip replacement [THR]) is a successful procedure and over 1 million procedures are performed every year worldwide. It is well recognised that acetabular cup orientation impacts directly on the mechanics of the joint and influences joint function postoperatively.^1–8^ The mechanical alignment approach, defined half a century ago, aims to achieve a predetermined positioning regardless of individual patient anatomy and is still adhered to by the majority of hip surgeons.^9–11^ More recently, the concept of combined femoral-acetabular anteversion has become popular with increased use of uncemented implants as well as taking into account the effect of lumbo-pelvic kinematics.^9,12–16^ Regardless of philosophy the consequences of acetabular component malposition include dislocation,^17,18^ increased wear,^9–21^ impaired muscle function,^22^ reduced range of motion (ROM),^23^ impingement,^23–25^ bearing-related noise generation,^26,27^ poor functional outcomes,^28^ limb-length discrepancy,^29^ and loosening and cup failure.^30–32^ Optimal acetabular cup orientation is commonly guided by aligning the cup within the Lewinnek safe zone of 30–50° inclination and 5–25° anteversion.^18^ However, achieving the intended acetabular cup orientation reliably intraoperatively remains a challenge for engineers and surgeons.^7^ Although surgeons aim to achieve optimal cup orientation a high variability has been observed in their ability to do so.^33–35^

A number of tools have been employed to help determine intraoperative component cup placement, including: mechanical alignment guides; digital and mechanical protractors; computational and robot assisted navigation systems; and inclinometers.^14,36–40^ The most common devices are mechanical alignment guides (MAGs). MAGs are cost effective but are designed to achieve a single prescribed angle which limits their usefulness for surgeons aiming to achieve an alternative angle. A majority of MAGs aim for a 45° operative inclination angle yield a radiographic inclination angle outside the safe zone owing to the influence of pelvic positioning in the lateral decubitus position.^41^ Furthermore, a MAG is primarily a passive visual reference for the surgeon, which relies on the ability of the surgeon to subjectively judge inclination without objective feedback, to achieve a pre-determined angle. As with the freehand technique, the application of MAGs, even in the hands of experienced surgeons, allows for significantly more error when trying to orient acetabular implants within the safe zone. Computer and robotic navigation systems offer the most accurate means to achieve optimal component placement but come with added complexity, potentially higher preoperative radiation exposure, the risk of pin site complications, considerable expense and a potential reduction in list productivity.

The use of an inclinometer is an attractive option as it can provide a more accurate means of measuring intra-operative acetabular component inclination than MAGs but is simpler, more efficient and more cost-effective than computer navigation. Over the last decade an increasing number of innovators have described techniques using inclinometer related devices to help surgeons achieve their desired inclination.^7,42^ Despite the recognition of the importance of accurate acetabular component placement, there are relatively few studies describing the use of inclinometer type devices. The aim of this systematic review was to assess the effectiveness of using an inclinometer in achieving the intended acetabular cup inclination angle and in reducing the angle variation.

## Methods

A systematic search of MEDLINE, Embase and Web-of-Science databases was performed. Both *in vitro* and *in vivo* studies looking at the accuracy of cup placement were included.

### Search protocol

The following search terms were used: (‘hip’ OR ‘hip replacement’ OR ‘hip arthroplasty’ OR ‘primary hip replacement’ OR ‘THR’ OR ‘THA’ OR ‘Acetabular cup Inclination’) AND (‘Inclinometer’). Titles and abstracts were screened for relevance. A citation search of the selected articles was performed to establish if further relevant articles were available. The full text of the selected studies was reviewed to assess for the inclusion criteria and methodology.

### Eligibility criteria

- Articles written in English
- Full text available
- Studies which compared an inclinometer method to a control group using an established conventional technique
- THA procedures performed in the lateral decubitus position
- Studies reporting angular measurement of inclination as an outcome (Both radiographic and operative inclination comparisons were included)
- Human studies (both *in vitro* and *in vivo*) (animal studies excluded).

### Data extraction

Papers meeting the inclusion criteria were reviewed to extract the relevant data. The data extracted included study design, sample size, cup placement method, cup inclination, adherence to safe zone (outliers), procedural time and dislocation rate.

### Analysis

A meta-analysis was not carried out as there was a lack of homogeneity in study methodology and reported outcomes. In particular, there were differences in target angles, safe zones, and measurement of radiographic inclination or operative inclination.

## Results

### Search results

In total 122 titles were screened. 107 were excluded either due to duplication or irrelevance. 15 abstracts were selected for review. 8 studies were identified that looked at the use of an inclinometer for measuring acetabular component inclination;^7,36,37,42–46^ of these 7 studies met the inclusion criteria.^7,36,37,42–45^ 2 studies were randomised control trials with level I evidence and the remaining studies were cohort studies with level III/IV evidence.^42,44^ 5 of the studies were clinical and the remaining 2 were experimental (1 cadaveric and 1 sawbone simulation). 5 studies compared radiographic inclination,^36,37,43–45^ and 2 compared operative inclination.^7,42^ 4 studies^36,37,43,44^ compared inclinometer to freehand techniques,^36,37,43,44^ 2 studies compared freehand, MAG and inclinometer techniques,^7,42^ and the remaining study compared MAG and inclinometer techniques.^45^ In total there were 16 cohorts; 7 using an inclinometer, 6 freehand, and 3 using a MAG. The detailed characteristics of the included studies and the data extracted are summarised in Table 1.

**Table 1.** Table showing the studies meeting the inclusion criteria and extracted data.

	Study type	Study description	Method	No. Surgeons	No. Hips	Male/ Female	Mean Age	Approach	BMI	Positioning	Target RI	Safe Zone	Mean (SD) Inclination	Range Inclination	% outliers	Procedure/Operative Time	*p*-value: comparison of means	*p*-value: comparison of outliers	*p*-value: procedure time	Dislocations	Comments
**Radiographic Inclination (RI)**																					
**Meermans et al.** ^37^	clinical	cohort	Freehand	3	100	38/62	65.9(9.9)	posterior	28.0(4.0)	Lat Decubitus	40	35-45	38.5(7.0)	22-60	28	52 mins	*p* = 0.80	*p* =0.002	*p* = 0.19	n/r	
			Inclinometer		100	34/66	64.7(9.0)	posterior	28.1(5.0)	Lat Decubitus	40	35-45	38.3(4.7)	27-51	10	50 mins				n/r	
**Darrith et al.** ^36^	clinical	cohort	Freehand	1	52	28/24	56.6.(6.2)	posterior	26.7(4.8)	Lat Decubitus	40 (30)*	30-50	46.5(6.3)	32.8-63.2	21	n/r	*p* = 0.004	*p* = 0.034	n/r	0	* 30 degrees OI target assuming 10 degrees greater for RI
			Inclinometer		68	36/32	57.5(6.5)	posterior	28.6(4.5)	Lat Decubitus	40 (30)*	30-50	42.9(7.0)	29.0-63.8	13	n/r				0	
**Vendittoli et al.** ^43^	cadaver	cohort	Freehand	14	50	1 male	n/r	Hardinge	medium size'	Lat Decubitus	40	30-55	44.4(11.4)	n/r	8	n/a	*p* = 0.83	*p* = 0.49	n/a	n/a	
			Inclinometer		50	1 male	n/r	Hardinge	medium size'	Lat Decubitus	40	30-55	42.2(3.8)	n/r	0	n/a				n/a	
**Vendittoli et al.** ^44^	clinical	RCT	Freehand	7	53	n/r	n/r	posterior or direct lateral	n/r	Lat Decubitus	40-49	30-55	42.7(6.7)	27.5-63.0	4	n/r	*p* = 0.56	*p* =0.536	n/a	n/r	
			Inclinometer		47	n/r	n/r	posterior or direct lateral	n/r	Lat Decubitus	40-49	30-55	43.6(6.8)	31.5-64.0	6	n/r				n/r	
**Pongkunakorn et al.** ^45^	clinical	cohort	MAG	1	41	20/21	51.1(8.6)	posterior	24.1 (4.2)	Lat Decubitus	40	30-50	46.3(6.7)	28.7-59.0	34	119(23) mins	*p* < 0.001	*p* = 0.001	*p* = 0.011	2	
			Inclinometer		41	22/19	54.5(7.9)	posterior	23.5 (3.8)	Lat Decubitus	40	30-50	40.9(3.8)	32.9-48.9	0	136(27) mins				0	
**Operative Inclination (OI)**																					
**O’Niell et al.** ^42^	clinical	RCT	Freehand	2	90	*	**	posterior	***	Lat Decubitus	35	32.5-37.5	32.9(2.9)	25.2-43.2	49	n/r	*p* < 0.001	*p* < 0.001	n/a	n/r	no significant difference in sex (p = 0.725)* or body mass index (BMI) (p = 0.298)** between groups. There was a a statistically significant difference in patient age (p = 0.034)***
			MAG		90	*	**	posterior	***	Lat Decubitus	35	32.5-37.5	33.7(1.9)	29.3-39.3	29	n/r	*p* = 0.023	*p* = 0.006	n/a	n/r	
			Inclinometer		90	*	**	posterior	***	Lat Decubitus	35	32.5-37.5	34.0(1.6)	27.5-37.3	12	n/r					
**Sykes et al.** ^7^	sawbone	cohort	Freehand	10 (10)	10	n/a	n/a	posterior	n/a	Lat Decubitus	*	+/- 2.5	6.2( 4.2)**	22.9-40.1***	78	4.5(2.67) secs	*p* = 0.001	n/r	*p* = 0.001	n/a	* Different target angles from 20°- 55° at 5° increments using each technique ** absolute mean error *** At 35° increment
			MAG		10	n/a	n/a	posterior	n/a	Lat Decubitus	*	+/- 2.5	3.8(3.3)**	31.4-41.3***	58	4.1(2.61) secs	*p* = 0.007	n/r	*p* = 0.001	n/a	
			Inclinometer		10	n/a	n/a	posterior	n/a	Lat Decubitus	*	+/- 2.5	0.6(0.5**	34.4-36.5***	0	5.9(3.97) secs				n/a	

n/r, not reported; n/a, not available; MAG, mechanical alignment guide; lat decubitus, lateral decubitus; OI, operative inclination; RI, radiographic inclination.

**Table 2.** Table showing the comparison of mean inclination angles with associated standard deviations and ranges for freehand, MAG, and inclinometer methods. The lower section showing the comparison of normalised values.

**Study**	**Target RI**	**Safe zone**	**Freehand**				**MAG**				**Inclinometer**			
			**Mean**	**SD**	**Range**		**Mean**	**SD**	**Range**		**Mean**	**SD**	**Range**	
					min	max			min	max			min	max
**Meermans et al.** ^37^	40	35-45	38.5	7.0	22.0	60.0					38.3	4.7	27.0	51.0
**Darrith et al.** ^36^	40 (30)*	30-50	46.5	6.3	32.8	63.2					42.9	7.0	29.0	63.8
**Vendittoli et al.** ^43^	40	30-55	44.4	11.4							42.2	3.8		
**Vendittoli et al.** ^44^	40-49	30-55	42.7	6.7	27.5	63.0					43.6	6.8	31.5	64.0
**Pongkunakorn et al.** ^45^	40	30-50					46.3	6.7	28.7	59.0	40.9	3.8	32.9	48.9
**O'Niell et al.** ^42^	35	32.5-37.5	32.9	2.9	25.2	43.2	33.7	1.9	29.3	39.3	34.0	1.6	27.5	37.3
**Sykes et al.** ^7^	35*	+/- 2.5	6.2	4.2	22.9	40.1	3.8	3.3	31.4	41.3	0.6	0.6	34.4	36.5
**Normalised Values**														
**Meermans et al.** ^37^			–1.5	7.0	–18.0	20.0					–1.7	4.7	–13.0	11.0
**Darrith et al.** ^36^			6.5	6.3	–7.2	23.2					2.9	7.0	–11.0	23.8
**Vendittoli et al.** ^43^			4.4	11.4							2.2	3.8		
**Vendittoli et al.** ^44^			–1.8	6.7	–17.0	18.5					–0.9	6.8	–13.0	19.5
**Pongkunakorn et al.** ^45^							6.3	6.7	–11.3	19.0	0.9	3.8	–7.1	8.9
**O’Niell et al.** ^42^			–2.1	2.9	–9.8	8.2	–1.3	1.9	–5.7	4.3	–1.0	1.6	–7.5	2.3
**Sykes et al.** ^7^			6.2	4.2	–12.1	5.1	3.8	3.3	–3.6	6.3	0.6	0.6	–0.6	1.5

MAG, mechanical alignment guide; SD, standard deviation; RI, radiographic inclination.

### Cup inclination

**Figure 1.**
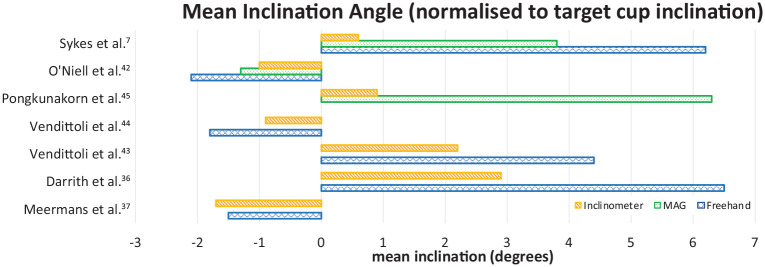
Bar chart showing normalised mean inclination angles for the 3 methods, freehand (blue/crosshatch), MAG (green/dot fill), Inclinometer (orange/parallel hatch).

Overall the use of an inclinometer resulted in a mean inclination angle closer to the target angle when compared to freehand and MAG techniques; all studies comparing radiographic inclination and 1 of 2 studies comparing operative inclination showed an improvement in the attainment of the optimal inclination. The standard deviations and ranges were larger for the freehand and MAG techniques. An overview of the mean cup inclination achieved using freehand, MAG, and inclinometer techniques with reported standard deviations and ranges is shown in Table 2 and Figure 1. Venditolli et al.^44^ found the difference in mean operative inclination between freehand and inclinometer cohorts was not significant (*p* = 0.49 and *p* = 0.536).^43,44^ All the remaining studies reported a statistically significant difference.

### Outliers

**Figure 2.**
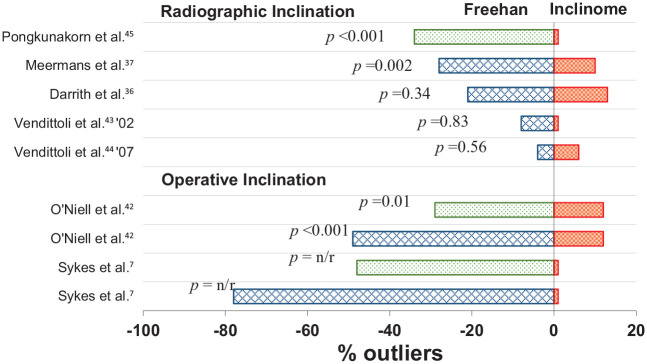
Bar chart representing the comparison of methods [freehand (blue/crosshatch), MAG (green/dot fill), inclinometer (red/parallel hatch)] with regard to the number of outliers (outliers as specified by referenced studies.

The use of an inclinometer resulted in a reduced number of outliers (inclination outside of specified safe zone) in all 3 comparisons to the use of a MAG; 2 were statistically significant,^7,42^ and 1 did not report significance (Figure 2).^45^ Of the 6 comparisons to freehand insertion all studies except Vendittolii et al.^44^ showed a reduction in outliers when an inclinometer was used; 3 were statistically significant,^36,37,45^ 2 were not significant.^43,44^ and 1 did not report significance.^45^

### Dislocation

Only 2 of the clinical studies reported the associated dislocations. Darrith et al.^36^ reported no dislocations in either freehand or inclinometer groups. Pongkunakorn et al.^45^ reported 2 dislocations in the freehand group both which they report being outside of the specified safe zones.

### Procedure duration

3 studies made a comparison of the surgical time associated with each of the methods in all the studies the use of an inclinometer was noted to require 2 and 7 minutes longer with regard to total procedure time in the clinical studies.^37,45^ The use of an inclinometer device was 1.4 and 1.8 seconds slower in sawbone simulations than freehand and MAG techniques respectively.^7^

## Discussion

Overall this review showed that in general there was a significant improvement in the accuracy of achieving the intended acetabular inclination angle as well as a significantly reduced risk of component placement outside of the safe zone when using an inclinometer over a MAG or freehand placement.

4 of the 6 studies showed a significant improvement in the accuracy of the cup inclination angle when an inclinometer was used when compared to a freehand technique; this was the case for all 3 cohorts comparing an inclinometer to the use of a MAG. All but 1 study showed that the use of an inclinometer reduced the number of outliers (outside of specified safe zone) when compared to freehand placement or placement with the assistance of a MAG. Of the 9 cohorts the significance for 2 was not reported but of those remaining only 1 did not show a significant reduction in outliers. This review included the comparison of both clinical and experimental studies looking at both operative and radiographic inclination. If the 5 clinical studies are assessed in isolation all except Vendittoli et al.^44^ showed a significant improvement in in accuracy and reduction in outliers with the use of an inclinometer.^36,37,42,44,45^

The term inclinometer has been used to describe the different devices used in the different studies. An inclinometer or clinometer is a device used for measuring angles of slope (or tilt), elevation, or depression of an object relative to gravitational orientation. The inclinometer designs described in the reviewed studies differ in that they employ different means of determining their orientation relative to gravity. 3 studies made use of mechanical devices. Darrith et al.^36^ used a simple bubble inclinometer placed on the acetabular insertion rod to measure inclination. The other studies used variations of gravity-actuated pendulums attached to the insertion rod.^43,44,46^ These inclinometers were scaled at different intervals ranging from 0° to 70° or 0° 180° and could be calibrated to set 0° as parallel to the insertion rod. Electronic devices positioned on the insertion rod can provide the surgeon with accurate digital readings of the operative inclination angle.^7,37,42^ Pongkunakorn et al.^45^ used the integrated inertial measurement unit of a smartphone with a level application to act as an inclinometer. The mechanical devices have the advantage of being sterilisable whereas the electronic devices needed to be placed in a sterile cover (e.g. camera drape used in arthroscopy). The devices described were used in conjunction with standard insertion instrumentation however, 2 studies described the use of custom brackets to attach their devices to the cup inserter.^7,45^

There was a lack of homogeneity between included articles regarding their methodology and reported outcomes. In particular, there were differences in target angles, safe zones, and measurement of radiographic inclination or absolute orientation. 2 measures were used to assess acetabular cup inclination: operative inclination (OI) and radiographic inclination (RI). OI refers to the angle of inclination of the acetabular cup in relation to the pelvic sagittal plane.^41,47,48^ RI refers to the angle between the plane of the cup opening and the inter-teardrop line on a postoperative anteroposterior standing radiograph.^49^ Radiographic inclination often differs from OI.^50^ RI is greater than OI owing to the influence of component anteversion on radiographic projection. Meermans et al.^37^ showed RI measurements 12.3° greater than the OI on average, regardless of whether or not cup placement was done freehand or with a protractor. Hill et al.^41^ similarly found a similar degree of deviation with RI measurements 13° greater than the OI on average. There are several potential factors that increase the tendency for discrepancies to occur between intraoperative and postoperative radiographic measurements of inclination. In the lateral decubitus position the pelvis has the propensity to tilt posteriorly resulting in a difference between the perceived and the actual OI.^44^ Additionally, smaller hip circumferences and certain hip support techniques have been found to be correlated with increased pelvic movement, compounding differences between RI and OI.^34,37^ Movement throughout the operation may cause errors in attempting to achieve an optimal angle of inclination.^42^ Additionally the process of impaction can result in discrepancies owing to pelvic movement during impaction as well as movement of the instrumentation. O’Neill et al.^42^ measured inclination before impaction, after the first impaction, and after the final impaction, respectively and found that, on average, the inclination angle decreased by 1.2° from the first impaction to the final impaction.

Regardless of method used; alignment of the acetabular cup within this safe zone is done under the assumption that anatomic landmarks of patients in lateral decubitus do not move intraoperatively and are uniform in position between patients.^47^ However, it has been found that not only does the pelvis move intraoperatively,^44^ but also that the degree of pelvic tilt in lateral decubitus varies widely between patients.^51^ Variations in posterior or anterior tilts results in variations in cup orientation, as adjustments for any additional retroversion or anteversion may not be recognised.^52^ Preoperative imaging may help with templating to minimise position error due to patient anatomic variation, but intraoperative pelvic movement still poses a problem in consistently achieving optimal cup orientation.

Proper patient positioning and levelling of the operating table is vital in reducing the incidence of acetabular cup malposition. In all studies, patients or cadavers were placed in the lateral decubitus position. To ensure standardised positioning of the patient, identical pubic and lumbosacral positioning devices were used for all the surgeries.^36,37,43,45^ Some groups also instructed the same medical assistant to position every patient.^36,42^ O’Neill et al.^42^ used a universal lateral positioner system in order to guarantee 3-point pelvic support. Pongkunakorn et al.^45^ implemented cross-table fluoroscopy to verify standardised patient positioning. Vendittoli et al.^43^ and Wilairatan et al.^46^ supported the pelvis of the cadaver at 90° to the plane of the operating table. Meermans et al.^37^ removed any patients from the study that the anaesthesiologist decided to place in Trendelenburg or reverse Trendelenburg. 4 studies performed the posterior approach,^7,36,37,42^ 1 the posterolateral,^45^ 1 the lateral,^43^ and 1 the lateral or posterior.^44^

Incorrect preoperative positioning of the pelvis and alterations in pelvic tilt during surgery are 2 major sources of acetabular malposition.^45^ Cross-table fluoroscopy can eliminate errors in preoperative pelvic positioning by allowing the surgeon to tilt the operating table in multiple planes so the fluoroscopic picture is symmetrical and orthogonal to the floor. Furthermore, a rotating C-arm ensures the orientation of the pelvis on the fluoroscopic image matches the preoperative image. Once the patient’s pelvis is correctly oriented in the lateral decubitus position, the smartphone application described by Pongkunakorn et al.^45^ can record this position as 0°. Any pelvic movement during the surgery can be detected by the smartphone application level and corrected for by turning the table to achieve the original 0°set point. These problems can be prevented by consistent, standardised positioning of the patient and table throughout the surgery which seems to be more likely if different levelling and calibration technologies are applied.

Inappropriate cup positioning has been associated with decreased stability, increased wear, impingement, and diminished function.^42^ Lewinnek et al.^18^ noted that the optimal position of the acetabular cup is 30-50° inclination and 5-25° anteversion. This was based on the finding that acetabular cups positioned outside these ranges of inclination and anteversion resulted in dislocation at a rate 4 times greater than positioning within these angle ranges.^18^ This has been referred to as the “safe zone” for positioning. Whether or not the Lewinnek safe zone is the gold standard target is still under contention.^45^ Abdel et al.^53^ found that a majority of THA-related dislocations were within the Lewinnek safe zone. As a result, other studies aimed to refine the safe zone for acetabular cup positioning. Danoff et al.^54^ proposed a safe zone with the Lewinnek angle of inclination of 30–50° and a narrower 10–25° of anteversion. Elkins et al.^55^ proposed a much narrower safe zone of 37.5–47.5° inclination and 12–22° anteversion. However, a narrowed safe zone is argued to be difficult for surgeons to consistently attain; surgeons who operate using the posterior approach have been found to attain the Lewinnek safe zone at a rate of only 60%.^35^

It is difficult to draw concrete conclusions regarding the specific benefit of using and inclinometer intra operatively due to variability between studies and the likely multifactorial considerations when determining the inclination of the acetabular component. However, based on the studies reviewed, the intraoperative use of an inclinometer was broadly shown to be a useful instrument in improving the surgeon’s ability to achieve their intended acetabular component inclination angle and reducing the incidence of straying from the safe zone. It should be noted that although the use of an inclinometer improved the acetabular component inclination angle; patient positioning and to a lesser extent ensuring that pelvis is stable intra-operatively are equally influential.

None of these studies provide long-term clinical data in large sample sizes to assess the impact of using inclinometer on patient outcomes and risk of hip dislocation. In addition, these devices only measure inclination and not the cup anteversion an equally important parameter contributing to implant performances.^56,57^ Further studies looking at the use of inclinometers to achieve accurate cup inclination intra operatively would prove useful if a multi-centre prospective study can demonstrate their useful in improving patient outcomes and reducing the number of outliers as well as complications related to cup malpositioning.
